# Structured telemonitoring reduces HbA1c and emergency visits in insulin-treated type 2 diabetes: a controlled cohort study in Ecuador’s public hospital

**DOI:** 10.3389/fcdhc.2026.1734589

**Published:** 2026-02-09

**Authors:** Angel Santillán, Edwin Ivan Travez Proaño, Ivanna Noemí Jaramillo Encalada, Patricio Alejando Abril López, Jennifer Tricallotis, Jaime David Acosta-España

**Affiliations:** 1 Hospital General Docente de Calderon (HGDC), Ministry of Health of Ecuador, Quito, Ecuador; 2 Doctorando en Ciencias Biomédicas del Instituto Universitario Italiano de Rosario (IUNIR), Rosario, Argentina; 3 Health Sciences Faculty, Universidad Internacional SEK (UISEK), Quito, Ecuador; 4 School of Medicine, Pontificia Universidad Católica del Ecuador, Quito, Ecuador; 5 Centro de Investigación para la Salud en América Latina (CISeAL), Pontificia Universidad Católica del Ecuador, Quito, Ecuador

**Keywords:** diabetes mellitus type 2, health services accessibility, insulin therapy, remote monitoring, telemedicine

## Abstract

**Background:**

Remote patient monitoring (RPM) has demonstrated potential to improve glycemic control in type 2 diabetes mellitus (T2DM), yet evidence from middle-income public health systems remains limited. This study evaluated the clinical impact of structured telemonitoring in insulin-treated T2DM patients at a public hospital in Ecuador.

**Methods:**

A prospective, controlled cohort study was conducted over a six-month period. Adults with insulin-treated T2DM and baseline HbA1c >8% were assigned to structured telemonitoring or standard care. The intervention included Bluetooth-enabled glucometers linked to the mySugr app, automated data uploads, and weekly clinical review. The primary outcome was the change in HbA1c; secondary outcomes included emergency visits and hospitalizations. Repeated measures ANOVA assessed HbA1c trajectories. Emergency visits were analyzed using Fisher’s Exact Test and Firth’s logistic regression.

**Results:**

Among 100 patients (50 per group), mean HbA1c decreased by 2.67% in the intervention group compared to 1.38% in the control group (p = 0.006). Emergency visits occurred in eight control patients and none in the intervention group (p = 0.006). Firth’s regression showed a non-significant odds ratio. No hospitalizations were reported. Direct cost savings totaled USD 9,660 over six months for the studied population and USD 174,000 annually, based on a 10% adoption rate (450 patients) over one year, using 2024 data from HGDC.

**Conclusion:**

Structured telemonitoring was associated with improved glycemic control and reduced utilization of acute care services. These findings support RPM feasibility in middle-income public health settings.

## 1 Introduction

Type 2 diabetes mellitus (T2DM) represents a significant global health challenge, ranking among the leading causes of mortality and disability worldwide (1). According to the IDF Diabetes Atlas 2025, approximately 589 million adults are currently living with diabetes, with projections estimating an escalation to 853 million by 2050 (2). The prevalence is disproportionately higher among older adults, with rates surpassing 20% in individuals over 65 years of age (2). Beyond clinical morbidity, diabetes imposes a staggering economic toll; global health expenditures reached USD 966 billion in 2023 and are anticipated to breach the USD 1 trillion threshold by 2045 (2).

In Latin America, diabetes affects between 8% and 13% of the population, accounting for roughly 13% of the total health expenditure within the region (3). Ecuador mirrors this trend, with prevalence estimates ranging from 1.7% to 7.9%, depending on the studied population (4, 5). Diabetes ranks as the fourth leading cause of mortality nationally (6), and the costs associated with care, particularly for patients undergoing insulin therapy, range from USD 800 to over USD 22,000 annually (7). These figures underscore the pressing need for scalable and cost-effective strategies aimed at enhancing disease management and reducing preventable complications.

Type 2 diabetes mellitus is largely preventable, and many of its complications, including diabetic ketoacidosis, retinopathy, nephropathy, and lower-limb amputations, are avoidable through timely intervention and sustained glycemic control (8, 9). Nevertheless, poor adherence, limited access to endocrinology services, and fragmented follow-up care contribute to elevated rates of preventable hospitalizations across the region, with estimates ranging from 4.3% to 15.3% (10).

Remote glucose monitoring reduces the need for frequent in-person consultations, decreasing clinical burden for both patients and providers. Telemonitoring facilitates continuous glucose monitoring, early detection of hypoglycemia and hyperglycemia, and prompt therapeutic adjustments, particularly beneficial for insulin-treated patients who require precise titration and frequent clinical oversight (11). The clinical imperative for these technologies was decisively accelerated by the COVID-19 pandemic, which transformed telemonitoring from an adjunctive option into an essential mechanism for continuity of care (12–14).

Recent meta-analyses and randomized controlled trials have demonstrated that RPM can significantly reduce HbA1c levels, enhance person living with diabetes satisfaction, and decrease emergency department visits (15–17). However, most evidence originates from high-income countries, and data regarding public health systems in low- and middle-income countries (LMICs) remain limited. In response to these gaps, the *Hospital General Docente de Calderón* (HGDC), a public secondary healthcare facility in Quito, Ecuador, has implemented a telemonitoring program for individuals with T2DM who are living with insulin-treated diabetes. This intervention integrates Bluetooth-enabled glucometers (Accu-Chek Instant), a mobile application (mySugr), and remote clinical review to support glycemic control and reduce acute care utilization. This study assesses the clinical impact of this program over six months, with a focus on HbA1c reduction, emergency visits, and reported outcomes among persons living with diabetes.

## 2 Materials and methods

### 2.1 Study design and setting

We conducted a prospective controlled cohort study at the outpatient facility of the *Hospital General Docente de Calderón* (HGDC) in Quito, Ecuador. According to Ecuador’s classification system, it is a second-level hospital with a complexity rating of 6. The study included a six-month follow-up period and compared outcomes between people living with DMT2 who received structured remote telemonitoring and those who received standard outpatient care without telemonitoring.

### 2.2 Participants

Eligible participants were adults aged 30–80 years with a confirmed diagnosis of T2DM, under insulin therapy or combined insulin–oral treatment, and with a baseline glycated hemoglobin (HbA1c) level of> 8%. Recruitment was conducted via consecutive sampling of the daily appointment logs at the HGDC Endocrinology outpatient clinic. Additional inclusion criteria for the intervention group included access to a smartphone compatible with the mySugr application and internet connectivity.

Exclusion criteria comprised persons living with diabetes type 1 diabetes, renal failure (glomerular filtration rate < 30 mL/min), pregnancy, severe comorbidities (e.g., advanced psychiatric disease, major visual impairment), or lack of family support for self-management. For the telemonitoring arm, access to a smartphone compatible with the mySugr app and internet connectivity was required (18).

### 2.3 Sample size and randomization

The sample size was pragmatically determined based on operational feasibility and recruitment capacity within the ambulatory diabetes unit of a public hospital. While no formal power calculation was used to define statistical significance thresholds, the target of 50 persons living with diabetes per group was informed by prior studies evaluating HbA1c change in telemonitoring interventions, which report clinically meaningful differences of ≥ 1.0 percentage point with standard deviations ranging from 1.5 to 2.0% (15, 16, 19). This sample size was deemed sufficient to detect directional trends and estimate effect sizes for future trials. Participants were allocated in a 1:1 ratio via random assignment administered by the treating physician at the time of enrollment. This method was selected to integrate seamlessly with the daily consultations of the diabetes unit.

### 2.4 Intervention: telemonitoring program

Persons living with diabetes in the intervention group received:

- Accu-Chek Instant^®^ glucometer linked via the mySugr app to the Accu-Chek Care^®^ platform.
- Four daily capillary glucose measurements (pre- and postprandial), uploaded automatically.
- Weekly data review by the diabetes care team (endocrinologist + diabetes nurse educator).
- Automatic alerts for hypoglycemia (<70 mg/dL) or hyperglycemia (>180 mg/dL) triggered a remote consultation, with insulin adjustment following the HGDC internal algorithm.

The control group received usual outpatient follow-up, including quarterly medical consultations and HbA1c testing, without telemonitoring, as it is part of the standard protocol of care that is in place HGDC.

### 2.5 Outcomes

Primary outcome: Change in HbA1c from baseline to 6 months.

Secondary outcomes: Emergency visits or hospitalizations due to acute diabetic complications (validated by hospital records), adults living with diabetes satisfaction with telemonitoring (standardized survey at 6 months), and exploratory projections of long-term complications and costs based on HbA1c reductions.

### 2.6 Data collection and laboratory procedures

HbA1c was measured at baseline (0 months), 3 months, and 6 months using standardized assays at the HGDC central laboratory. Demographic and clinical baseline data were obtained from medical records. Satisfaction survey data for the Diabetes Treatment Satisfaction Questionnaire modality status were collected at the end of follow-up in the intervention group (20). Sadly, because of missing data from the survey, Diabetes Treatment Satisfaction Questionnaire modality status was not included in this study. The response rate among participants was extremely low, leading to substantial missing data. To maintain the integrity of the dataset and prevent bias from imputation or analysis of incomplete records, we decided to exclude this survey data from the final analysis.

### 2.7 Statistical analysis

Continuous variables were expressed as mean ± SD or median (IQR). Within-group comparisons were analyzed using paired t-tests or Wilcoxon signed-rank tests, and between-group differences were analyzed using independent t-tests or Mann–Whitney U tests. Repeated measures were assessed using linear mixed-effects models to account for time and group interaction, allowing us to assess the intervention’s effect while accounting for individual variability. Proportions were compared with the Chi-square test. Results were reported as mean difference with 95% confidence intervals. Significance was set at p < 0.05.

### 2.8 Economic evaluation methods

We conducted a retrospective cost-effectiveness analysis from the hospital provider perspective to assess the economic impact of a telemonitoring intervention for insulin-treated diabetic persons. The analysis was based on a six-month implementation involving 50 adults living with diabetes. Unit costs for outpatient diabetes consultations of moderate complexity with a specialist were obtained from the official list of healthcare private and public service prices provided by the Ministry of Health of Ecuador, which range between 13.5 and 20.0 USD consultation. Telemonitoring-associated savings were calculated using a fixed monthly reduction of 32.2 USD per person living with diabetes in direct care costs. Acute care costs were modeled using an estimated unit cost of $280.0 to $300.0 per emergency visit or hospitalization related to diabetes complications.

For scalability modeling, anonymized institutional data from HGDC indicated an annual volume of approximately 4,500 consultations for persons living with diabetes treated with insulin. A 10% adoption scenario was applied, corresponding to 450 people living with diabetes receiving telemonitoring over a 12-month horizon. All monetary values were expressed in 2024 USD without discounting, and sensitivity analyses were conducted across ranges of consultation costs (13.5–20.0 USD), hospitalization cost estimates (280.0–300.0 USD), and adoption rates (5–25%). All data were anonymized prior to analysis, and the methodological framework adhered to CHEERS 2022 guidelines for health economic evaluations.

### 2.9 Ethical considerations

The study received approval from the HGDC Institutional Ethics Committee, protocol number CEISH-HGDC-2023-003. All participants signed informed consent before joining. Data confidentiality and the privacy of persons living with diabetes were protected in accordance with institutional protocols.

## 3 Results

### 3.1 Study population

A total of 100 persons living with insulin-treated type 2 diabetes mellitus (T2DM) were enrolled: 50 in the control group and 50 in the intervention group. No missing data were reported for any primary or secondary outcomes. The mean age was 60.2 ± 11.1 years in the control group and 55.7 ± 10.7 years in the intervention group. Gender distribution was balanced: 34 females and 16 males in the control group, and 33 females and 17 males in the intervention group (**Table 1**).

**Table 1:** Baseline demographic characteristics, clinical status, and comparative outcomes at 6 months for the Control (n=50) and Intervention (n=50) groups.

Characteristic	Control N = 50^1^	Intervention N = 50^1^	p-value^2^
Age (Years)	60.22 ± 11.15	55.72 ± 10.72	0.042
Female Gender			>0.9
Female	34 (68%)	33 (66%)	
Male	16 (32%)	17 (34%)	
Baseline HbA1c (%)	10.25 ± 2.25	9.82 ± 1.74	0.3
Month 6 HbA1c (%)	8.87 ± 1.72	7.58 ± 1.31	<0.001
Change in HbA1c (Delta)	-1.38 ± 2.54	-2.24 ± 1.60	0.046
Emergency Dept. Visits	8 (16%)	0 (0%)	0.006

^1^Mean ± SD; n (%).

^2^Welch Two Sample t-test; Fisher’s exact test.

Data are presented as mean ± standard deviation (SD) for continuous variables and n (%) for categorical variables. P-values were calculated using independent t-tests for continuous data and Fisher’s Exact Test for categorical data. Abbreviations: HbA1c, glycated hemoglobin.

### 3.2 Glycemic control (primary outcome)

**Figure 1:**
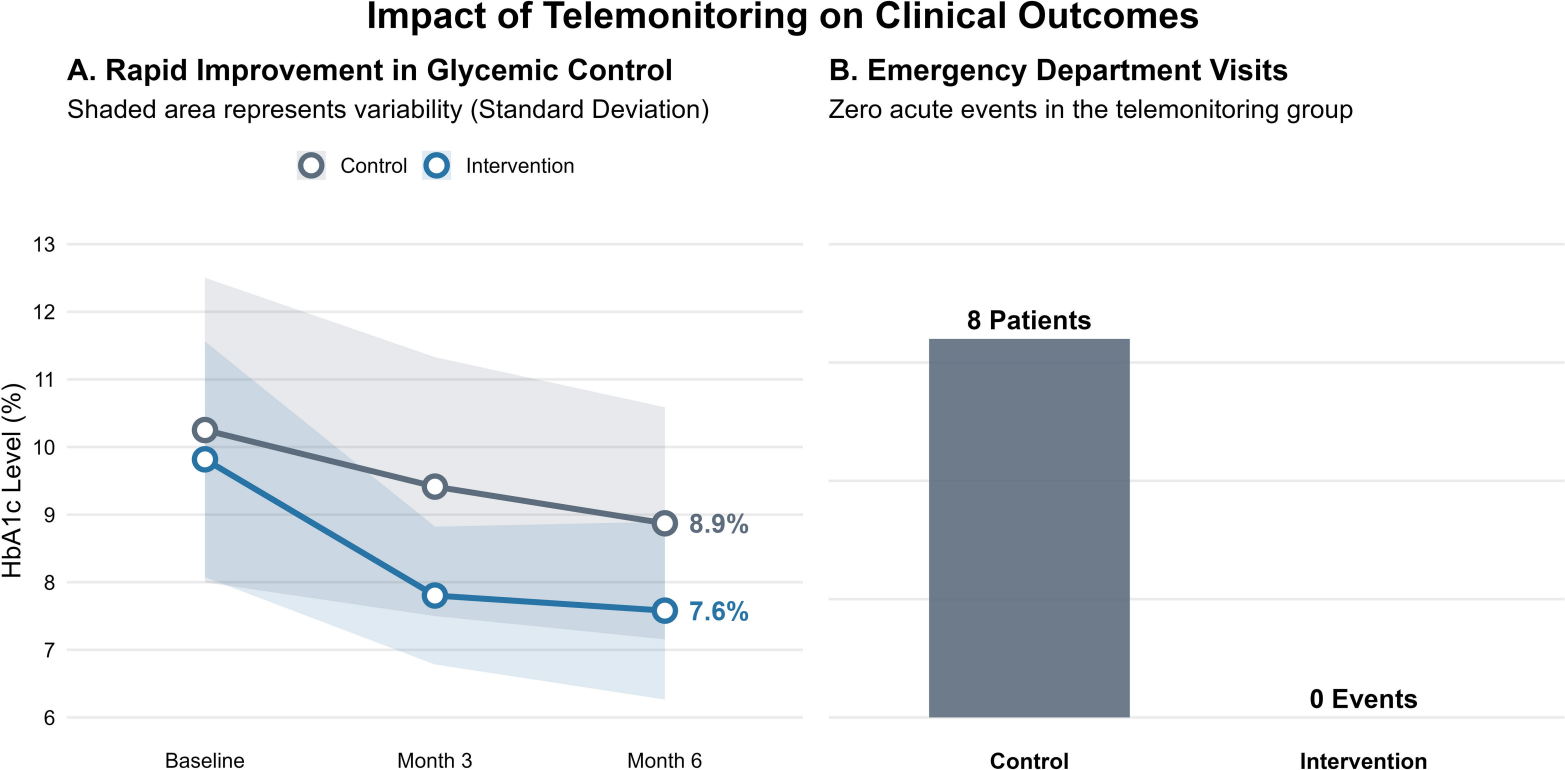
Impact of Structured Telemonitoring on Glycemic Control and Acute Care Utilization. **(A)** Longitudinal trajectory of HbA1c levels over the 6-month follow-up period. The intervention group (blue line) demonstrated a rapid and sustained reduction in HbA1c compared to the control group (grey line). The shaded ribbons represent the standard deviation (SD) to visualize data variability. By month 6, the telemonitoring group achieved a mean HbA1c of 7.6% vs. 8.9% in controls (p < 0.001). **(B)** Frequency of emergency department visits. The bar chart highlights the complete absence of acute diabetes-related events (0 visits) in the telemonitoring group, compared to 8 events (16% of patients) in the control group (Fisher’s Exact Test, p = 0.006).

Baseline HbA1c was 10.3% ± 2.25 in the control group and 9.82% ± 1.74 in the intervention group (**Figure 1A**). At 3 months, HbA1c decreased to 9.41% ± 1.91 in controls and 7.80% ± 1.02 in the intervention group. At 6 months, HbA1c reached 8.87% ± 1.72 in controls and 7.58% ± 1.31 in the intervention group. Repeated measures ANOVA revealed a significant time versus group interaction (F [1.67, 163.79] = 5.87, p = 0.006, η²_p_ = 0.057), indicating that HbA1c trajectories differed between groups. The main effect of time was also significant (F [1.67, 163.79] = 57.48, p < 0.001, η²_p_ = 0.370), as was the between-group effect (F(1, 98) = 16.6, p < 0.001, η²_p_ = 0.145). *Post hoc* comparisons (Tukey-adjusted) showed that the intervention group achieved significantly greater HbA1c reductions at 3 months (mean difference = −2.448%, p < 0.001) and 6 months (mean difference = −2.671%, p < 0.001) compared to controls. Within-group reductions were also significant for the intervention group at both time points (p < 0.001), while the control group showed modest but significant change at 6 months (mean difference = −1.379%, p < 0.001). Emergency Visits (Secondary Outcome) Emergency visits occurred in 8 persons living with diabetes (16%) in the control group and in 0 persons living with diabetes in the intervention group. Fisher’s Exact Test confirmed a statistically significant difference (p = 0.006), with a relative risk of 0.84 (95% CI: 0.744–0.948) and an odds ratio of 0.0495 (95% CI: 0.00278–0.883), calculated using the Haldane-Anscombe correction. Binomial logistic regression was attempted but failed due to complete separation (zero events in the intervention group). Firth’s penalized logistic regression was applied to correct for this. The model yielded an odds ratio near unity (OR ≈ 1.00, 95% CI: 0.00–exp (5.23), p = 1.00), indicating no statistically significant effect, though the absence of events in the intervention group suggests a potential clinical benefit.

### 3.3 Hospitalizations

No hospitalizations occurred in either group during the 6-month follow-up period. Days hospitalized remained at zero across all participants. No statistical analysis was carried out due to a lack of variance.

### 3.4 Emergency visits (secondary outcome)

Emergency visits occurred in 8 persons living with diabetes (16%) in the control group and in 0 persons living with diabetes in the intervention group (**Figure 1B**). Fisher’s Exact Test confirmed a statistically significant difference (p = 0.006), with a relative risk of 0.84 (95% CI: 0.744–0.948) and an odds ratio of 0.0495 (95% CI: 0.00278–0.883), calculated using the Haldane-Anscombe correction. Binomial logistic regression was attempted but failed due to complete separation (zero events in the intervention group). Firth’s penalized logistic regression was applied to correct for this. The model yielded an odds ratio near unity (OR ≈ 1.00, 95% CI: 0.00–exp(5.23), p = 1.00), indicating no statistically significant effect, though the absence of events in the intervention group suggests a potential clinical benefit.

### 3.5 Economic evaluation methods

In this study, 50 people living with insulin-treated diabetes received care via telemonitoring over a six-month period. According to the official healthcare service price list from the Ministry of Health of Ecuador, a moderate-complexity outpatient diabetes consultation with a specialist costs between USD 13.50 and USD 20 per adult living with diabetes. Telemonitoring resulted in a direct cost savings of approximately USD 32.20 per person living with diabetes per month, totaling USD 9,660 over the six-month period. During this period, no adult with diabetes required emergency care or hospitalization, thus avoiding additional costs estimated at USD 280–300 per event. This demonstrates the program’s efficiency in reducing acute complications. Extrapolating these findings to the anonymized data from the HGDC for 2024, where about 4,500 outpatient consultations are for insulin-treated persons, a telemonitoring program with a 10% adoption rate (450 persons) could potentially save nearly USD 174,000 annually, including outpatient visits and the prevention of hospitalizations. These results emphasize that, beyond being cost-effective in smaller groups, telemonitoring is both scalable and sustainable from the healthcare provider’s perspective.

## 4 Discussion

This controlled cohort study demonstrates that structured telemonitoring is associated with significantly greater reductions in HbA1c over six months compared to standard outpatient care among insulin-treated persons with type 2 diabetes mellitus (T2DM). The intervention group achieved a mean HbA1c reduction of −2.67%, while the control group showed a reduction of −1.38%. Repeated-measures ANOVA confirmed a significant time-by-group interaction (F = 5.87, p = 0.006, η²p = 0.057), indicating that the trajectory of glycemic control differed significantly between groups. These results are consistent with prior studies reporting improvements in HbA1c levels in RPM interventions, although the magnitude observed here exceeds the average reductions reported in meta-analyses of similar programs (21, 22).

The intervention’s impact appears particularly relevant in the context of Ecuador’s public health system, where access to endocrinology services is limited, and insulin titration is often delayed. The RPM model, as implemented, combining Bluetooth-enabled glucometers, automated data uploads, and weekly clinical reviews, may have facilitated more timely therapeutic adjustments (23, 24). While causality cannot be confirmed in a non-randomized design, the consistency of HbA1c reductions across time points and the absence of missing data strengthen the internal validity of the findings.

Emergency visit data further support the clinical relevance of the intervention. Eight persons living with diabetes in the control group experienced emergency visits, whereas none in the intervention group did. Fisher’s Exact Test yielded a statistically significant difference (p = 0.006), with an odds ratio of 0.0495 (95% CI: 0.00278–0.883) and a relative risk of 0.84 (95% CI: 0.744–0.948). Although logistic regression failed due to complete separation, and Firth’s correction yielded a non-significant result, the raw contingency data suggest a potential protective effect. However, given the small number of events and lack of hospitalizations in either group, these findings should be interpreted cautiously and warrant replication in larger samples.

Telemonitoring of insulin-treated diabetes persons proved both cost-effective and clinically efficient, reducing acute events and generating substantial savings. These findings support its scalability and sustainability within hospital-based care models.

Several limitations must be acknowledged. First, although the protocol specified mixed-effects modeling, only repeated measures ANOVA was applied. While appropriate for balanced data, ANOVA does not account for individual-level variability or potential missingness, which mixed models are designed to handle. Second, the sample size was pragmatically determined and not powered for secondary outcomes, limiting the precision of emergency visit estimates. Third, the exclusion of persons without smartphone access may introduce selection bias, potentially limiting generalizability to digitally underserved populations.

Successful implementation required overcoming specific barriers. Initial challenges included low digital literacy among older participants, which necessitated simplified training by the clinical staff. Furthermore, sporadic internet connectivity in certain areas highlighted the need for robust offline data syncing capabilities.

Finally, this study demonstrates that structured telemonitoring can improve glycemic control and reduce acute care use among insulin-treated patients in a public hospital setting. Yet, its single-center design in a tertiary hospital with specialist oversight limits generalizability, particularly for resource-constrained primary care or rural areas with unstable connectivity. Even so, the intervention’s simplicity and seamless integration into existing workflows highlight its potential for broader adaptation. Future work should employ mixed-effects modeling, evaluate long-term outcomes, and assess cost-effectiveness to guide policy and scale-up across diverse healthcare system.

## 5 Conclusion

Structured telemonitoring, when integrated into routine outpatient care, was associated with significantly greater reductions in HbA1c and no recorded emergency visits over six months among insulin-treated persons living with type 2 diabetes in a public hospital setting. These findings suggest that remote monitoring can deliver clinically meaningful benefits in glycemic control and acute care prevention, even within resource-constrained environments. The absence of emergency visits further reinforces the program’s cost-effectiveness by preventing high-cost acute care events. While the intervention’s simplicity and scalability support its feasibility for broader implementation, future studies should incorporate mixed-effects modeling, assess long-term outcomes, and evaluate directly cost-effectiveness to inform sustainable integration into national diabetes program.

## 6 Limitations

This study was conducted within the operational constraints of a public hospital in a middle-income country, which shaped both its design and analytical approach. Although the protocol specified mixed-effects modeling to account for repeated measures and individual-level variability, the final analysis relied on repeated-measures ANOVA due to the dataset’s balanced structure and the availability of accessible statistical platforms. While valid under these conditions, ANOVA does not account for random effects or intra-subject correlation, which can limit the precision of longitudinal estimates. Additionally, the sample size was pragmatically determined based on recruitment feasibility rather than formal power calculations, which may have reduced the study’s sensitivity to detect differences in secondary outcomes, such as emergency visits.

The exclusion of persons without smartphone access introduces a selection bias that may limit generalizability to digitally underserved populations. Furthermore, the complete absence of emergency visits in the intervention group led to separation in logistic regression, requiring Firth’s correction and yielding wide confidence intervals. Finally, although long-term projections of complication risk and cost savings were outlined in the protocol, they were not performed due to the lack of validated local cost data and modeling infrastructure. These limitations reflect the realities of conducting implementation research in constrained settings, but they also highlight the feasibility and potential impact of telemonitoring in public-sector diabetes care.

Additionally, a limitation regarding the assessment of digital adherence must be noted. Although the study protocol included a survey to evaluate patient engagement with the application and glucometer, the completion rate was insufficient for reliable analysis. To ensure data quality and avoid bias from missing values, these survey results were excluded. Future studies should prioritize automated backend logging of app usage over self-reported surveys to guarantee data completeness.

## Data Availability

The original contributions presented in the study are included in the article/supplementary material. Further inquiries can be directed to the corresponding author.
